# Anomalous Origin of the Right Coronary Artery From the Ascending Aorta Above the Left Sinus of Valsalva

**DOI:** 10.7759/cureus.13845

**Published:** 2021-03-12

**Authors:** Ramses Ramirez Damera, Muhammad I Khan, Kay T Khine, Neelima Katukuri

**Affiliations:** 1 Graduate Medical Education - Department of Internal Medicine, University of Central Florida College of Medicine/HCA Healthcare Greater Orlando, Orlando, USA; 2 Department of Cardiovascular Medicine, Orlando Veterans Affairs Medical Center, Orlando, USA; 3 Department of Medical Education, University of Central Florida College of Medicine, Orlando, USA

**Keywords:** coronary anomaly, anomalous right coronary artery, ascending aorta, coronary angiography

## Abstract

Congenital anomalous origin of the coronary arteries is a rare but well-described cause of myocardial ischemia and sudden cardiac death. Anomalous origin of the right coronary artery from the ascending aorta is an extraordinarily rare occurrence. We report a case of anomalous origin of the right coronary artery from the ascending aorta posteriorly above the left sinus of Valsalva found during coronary angiography for evaluation of newly diagnosed cardiomyopathy.

## Introduction

Anomalous origin of the coronary vessels (AOCV) is reported to occur at a rate of 0.28% and 1.3% in autopsy series and angiographic series respectively [1-4]. Anomalous origin of the right coronary artery (RCA) from the ascending aorta is an extraordinarily rare occurrence, comprising only 0.04 to 0.15% of all reported coronary anomalies. Early recognition of AOCV is crucial as it may lead to chest pain, dyspnea, acute coronary syndrome (ACS), syncope, and even sudden cardiac death (SCD) [5,6]. Anomalous coronaries have also been recognized as one of the most common causes of SCD in young athletes [7]. We report a case of anomalous origin of the right coronary artery from the ascending aorta posteriorly above the left sinus of Valsalva found during coronary angiography for evaluation of newly diagnosed cardiomyopathy.

## Case presentation

**Figure 1:**
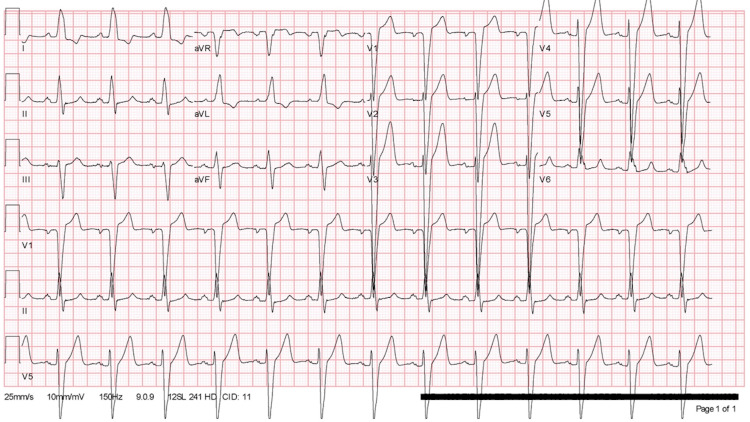
Electrocardiogram revealing a left bundle branch block, but no other abnormalities.

A 56-year-old gentleman presented to the cardiology clinic for evaluation of new-onset cardiomyopathy. He reported intermittent atypical chest pain, exertional dyspnea, and fatigue for several months. His medical history was significant for chronic left bundle branch block (LBBB) (Figure 1), tobacco, and alcohol abuse. His chart review revealed two previous pharmacologic nuclear stress tests; the first one was done three years prior to the presentation which revealed a small fixed antero-apical perfusion defect, and a repeat stress test a few months prior to his visit which then showed a large, fixed defect in the anteroseptal and inferoseptal wall. The patient subsequently underwent transthoracic echocardiography which revealed a reduced left ventricular ejection fraction of 40-45%, global hypokinesis with regional variation, and septal dyskinesia which was attributed to the LBBB, and no significant valvular abnormalities were seen. Consequently, the patient was started on guideline-directed medical therapy which included a beta-blocker, angiotensin-converting enzyme inhibitor (ACE-I), and was then referred for an elective coronary angiography to determine whether this was the result of ischemic heart disease.

At the patient’s visit to our cardiology clinic, his vital signs were within normal limits, with an unremarkable complete blood count and comprehensive metabolic panel. Coronary angiography was performed via the right radial approach, initial evaluation of the left coronary circulation was unremarkable (Video 1); however, upon trying to engage the RCA, the cardiologist was unable to recognize a take-off point within the coronary sinus. Interestingly, during the delayed images, a vessel in the distribution of the RCA was seen, but its origin appeared to be in the ascending aorta, above the coronary sinus of Valsalva (Video 2).

**Video 1:** Coronary angiography revealing left coronary circulation without any evidence of coronary artery disease.

**Video 2:** Aortogram showing a vessel in the RCA distribution (red arrow) during delayed filling. RCA: Right coronary artery

Shortly after, the patient developed severe radial artery spasm, so the interventionist had to change to a femoral approach. With a right femoral approach, successful cannulation of the RCA was achieved, confirming the anomalous origin of the RCA, originating from the ascending aorta above the left sinus of Valsalva (Video 3). No significant coronary artery disease was visualized and no interventions were performed. The patient was managed medically with beta-blocker and ACE-I therapy. A cardiac MRI was ordered but unfortunately, the patient was lost to follow-up.

**Video 3:** Coronary angiography revealing the anomalous origin of the RCA, from the ascending aorta above the left coronary sinus of Valsalva. RCA: Right coronary artery

## Discussion

AOCV are not routinely seen during clinical practice, so it is important to recognize them as some anomalies have serious consequences including but not limited to angina, ACS, and even SCD. Anomalous right coronary artery originating above the left sinus of Valsalva is a very rare subtype with limited case reports describing its existence. The pathophysiology behind its complications is still unclear, but most experts believe that it is associated with the mechanical compression of the RCA by the great vessels. In addition, its abnormal angulation at the take-off point, along with the more tortuous course of the proximal portion of the vessel can accelerate the rate of atherosclerosis [8]. Due to the scarce literature, there is no clear association with any specific process or an expected disease course. A complication directly linked to this subtype of AOCV was reported by Tarhan et al. where transection of the anomalous RCA occurred during surgical aortic valve replacement [9]. Cannulation of anomalous RCA may prove to be difficult which requires prompt recognition and adjustment of the access vector during coronary angiography [10]. The management of these patients remains a matter of debate, especially since there are no official guidelines published. Most experts tailor treatment based on the presence of symptoms and the anatomic anomaly in question. The management for anomalous right coronary artery originating above the left sinus of Valsalva is mainly based on observations and expert's opinion since the literature consists of case reports [11]. 

## Conclusions

Anomalous right coronary artery originating above the left sinus of Valsalva is a very rare congenital entity. However, its early recognition is crucial as it can cause myocardial ischemia, ventricular arrhythmias, and even SCD. These complications are thought to occur as a result of extrinsic compression of the RCA by the neighboring vessels and as a result of anatomic changes that occur to the vessel's origin, course, and vascular flow. The management of patients with this subtype of AOCV remains a matter of debate. Most clinicians base their judgment on the presence of symptoms or complications associated with the individual anomaly. Interestingly, an existing case report of this coronary anomaly report associated cardiomyopathy, whether this is an incidental finding or associated feature, has not yet been determined. Prospective data collection is essential to establish this possible correlation.
